# Real-world effectiveness and safety of ofatumumab in relapsing-remitting multiple sclerosis: Insights from naïve and switch patients

**DOI:** 10.1016/j.neurot.2025.e00724

**Published:** 2025-09-15

**Authors:** Clara G. Chisari, Salvatore Lo Fermo, Salvatore Iacono, Giuseppe Schirò, Francesca Ruscica, Sabrina Realmuto, Sebastiano Bucello, Paolo Ragonese, Giuseppe Salemi, Francesca Matta, Simona Toscano, Salvatore Cottone, Luigi Maria Edoardo Grimaldi, Francesco Patti

**Affiliations:** a Department of Medical and Surgical Sciences and Advanced Technologies "G.F. Ingrassia", University of Catania, Catania, Italy; b UOS Sclerosi Multipla AOU Policlinico G Rodolico-San Marco, University of Catania, Catania, Italy; c Neurology and Multiple Sclerosis Center, Unità Operativa Complessa (UOC), Foundation Institute "G. Giglio", Cefalù, PA, Italy; d Department of Biomedicine, Neurosciences and Advanced Diagnostics, University of Palermo, PA, Italy; e Multiple Sclerosis Centre, Neurology Unit and Stroke Unit, AOOR "Villa Sofia-Cervello", PA, Italy; f Multiple Sclerosis Center, "E. Muscatello" Hospital - ASP8, Augusta, SR, Italy; g Department of Neurology, Ospedale Garibaldi Centro, Catania, Italy; h A.R.N.A.S. CIVICO, PA, Italy

**Keywords:** Multiple sclerosis, Ofatumumab, Switch

## Abstract

Ofatumumab (OFA), a fully human anti-CD20 monoclonal antibody, has shown promising efficacy in treating relapsing multiple sclerosis (RMS) by depleting B cells and reducing disease activity. This real-world, prospective, multicenter study evaluated the effectiveness and safety of OFA in treatment-naïve patients and those transitioning from other disease-modifying therapies (DMTs), including natalizumab (NTZ). RRMS patients initiating OFA at seven MS centers in Sicily and treated for at least 12 months were analyzed. Outcomes included annualized relapse rates (ARR), Expanded Disability Status Scale (EDSS), and the percentage of patients free from relapse, MRI activity, and confirmed EDSS worsening (CEW). Of 213 patients, 66 (30.9 ​%) were naïve and 147 (69.1 ​%) were switchers. At 12 months, both groups showed comparable CEW-free (93.9 ​% vs. 93.8 ​%), relapse-free (92.4 ​% vs. 93.2 ​%), and MRI activity-free (84.8 ​% vs. 85.0 ​%) proportions. Within the high-efficacy group, NTZ-switchers showed significantly better MRI outcomes than those switching from other agents, while CEW-free and relapse-free rates remained similar. OFA was well tolerated with no serious adverse events. Predictors of non-response included high baseline MRI activity, disease duration >10 years, and prior NTZ and non-NTZ high-efficacy DMTs. These findings support OFA as a safe and effective option for RRMS across patient subtypes.

## Introduction

### Background on MS and the rationale for early high-efficacy treatment

Multiple Sclerosis (MS) is a chronic, autoimmune disorder characterized by inflammation, demyelination, and axonal damage within the central nervous system (CNS). It is a leading cause of neurological disability in young adults, with a global prevalence estimated at 2.8 million people [1]. The disease typically presents in a remitting form (RMS) in about 85 ​% of patients, characterized by episodes of neurological dysfunction (relapses) followed by periods of partial or complete recovery [2]. The pathogenesis of MS involves a complex interplay between genetic susceptibility and environmental factors, leading to an aberrant immune response directed against CNS components [3]. This process is facilitated by the infiltration of autoreactive lymphocytes, particularly T cells and B cells, across the blood-brain barrier (BBB) [4]. These immune cells orchestrate a cascade of inflammatory events, including the activation of macrophages and microglia, and the release of pro-inflammatory cytokines and chemokines. Recent advances have highlighted the critical role of B cells in MS pathogenesis [3,5]. B cells contribute to the disease not only through the production of autoantibodies but also by presenting antigens and secreting pro-inflammatory cytokines [6]. This understanding has paved the way for B cell-depleting therapies, which have shown significant promise in reducing disease activity and progression [7].

### CD20-targeting monoclonal antibodies and ofatumumab

Studies on ocrelizumab, rituximab and ublituximab reporting their efficacy in reducing relapse rates and MRI activity support the rationale that effectively targeting pathogenic B-cell populations is able to reduce inflammatory activity and minimizing clinical relapses [[8], [9], [10], [11], [12]]. Among these, Ofatumumab (OFA) has emerged as a promising agent due to its unique route of administration and favorable safety profile. OFA is a fully human monoclonal antibody targeting the CD20 molecule on B cells, leading to their depletion [13,14]. Initially developed for the treatment of B-cell malignancies, OFA has been repurposed for MS therapy due to its potent B cell-depleting effects. OFA binds to a unique epitope on the CD20 molecule, distinct from other anti-CD20 antibodies such as rituximab, ocrelizumab, and ublituximab [10,12,15,16]. This binding induces potent B cell lysis through mechanisms including complement-dependent cytotoxicity (CDC) and antibody-dependent cell-mediated cytotoxicity (ADCC) [17,18]. By depleting CD20-positive B cells, OFA disrupts their pathogenic role in antigen presentation, cytokine production, and autoantibody formation, thereby attenuating the inflammatory processes underlying MS [13]. The efficacy and safety of OFA in MS have been demonstrated in several clinical trials. The Phase III ASCLEPIOS I and II trials were pivotal in establishing OFA as an highly effective treatment for RMS. These studies compared OFA with teriflunomide and found that OFA significantly reduced annualized relapse rates (ARR), the number of new or enlarging MRI lesions, and the risk of confirmed disability progression [19]. More recently, real-world data on anti-CD20 therapies, including OFA, have increasingly supported their role in achieving disease control in RMS [20]. Moreover, previous studies evaluating CD20-depleting therapies, including rituximab, ocrelizumab, OFA, and ublituximab have emphasized the role of B-cell depletion in controlling disease progression [[10], [11], [12],^19^,^21^].

### Real-world evidence and the need for more data

Despite promising results from randomized controlled trials, real-world data on the effectiveness across diverse patient populations, especially those with complex therapeutic histories are to date lacking. Particularly, differences in treatment response between naïve patients and switchers may highlight distinct immunological or disease mechanisms, as well as the influence of prior therapies on disease activity and outcomes. Investigating these differences can inform personalized treatment strategies, optimize sequencing of therapies, and provide evidence for clinicians to better manage patient-specific challenges.

In addition, patients switching from natalizumab (NTZ) represent a particularly intriguing subgroup. NTZ is a highly effective therapy but is associated with risks such as progressive multifocal leukoencephalopathy (PML), often necessitating discontinuation despite ongoing efficacy [22]. Understanding how these patients respond to OFA, in terms of disease control and safety, is critical for guiding treatment decisions.

### Aim of the study

This study aims to investigate the effectiveness of OFA in a real-world setting, focusing on differences between treatment-naïve patients and those transitioning from other DMTs. Additionally, it aims to analyze clinical outcomes within the cohort previously treated with NTZ. By capturing real-life treatment scenarios, our study provides valuable insight into the positioning of OFA in the therapeutic algorithm of RMS, particularly in the context of treatment switching and escalation strategies.

## Materials and methods

### Study population

This prospective multicenter real-world study was conducted across seven MS centers in Sicily, Italy, from August 1, 2022 to January 1, 2025. The study included all consecutive patients diagnosed with RMS who initiated treatment with OFA and were treated for at least 12 months. Inclusion criteria comprised a confirmed diagnosis of RMS according to the 2017 revision of the McDonald criteria and eligibility for OFA treatment based on national and European guidelines [23]. Patients with incomplete follow-up data or treatment discontinuation were excluded.

### Data collection

Demographic and clinical data were collected at baseline, 6 (T6), and 12 (T12) months after OFA initiation. Expanded Disability Status Scale (EDSS) was measured at baseline, 6 (T6) and 12 months (T12) after OFA initiation, in order to assess changes in disability progression. ARR were also calculated before and during OFA treatment.

Documented treatment history was categorized as naïve (no prior DMT use) or switch (transitioning from other therapies). Data about Magnetic Resonance Imaging (MRI) (number of T2 lesions, number of new or enlarging T2 lesions, and gadolinium-enhancing lesions) were collected at baseline and at each time point. All treatment-emergent adverse events (AEs) were recorded, including infection rates, injection-related reactions, and other safety concerns.

### Outcomes

Primary outcomes included: changes in disease activity (ARR and MRI activity) and disability progression, in terms of confirmed EDSS worsening (CEW) from baseline to 12 months after OFA initiation. CEW was considered as ≥1.0-point increase from baseline if the baseline EDSS is ​≤ ​5.5, or ≥0.5-point increase if the baseline EDSS is ​> ​5.5 [24].

We also assessed the proportion of patients achieving No Evidence of Disease Activity 3 (NEDA-3) status at T12, considering: the absence of clinical relapses, no confirmed disability progression, and no new or enlarging T2 lesions on MRI [25].

Secondary outcomes included safety profile, and differences in terms of efficacy and safety outcomes between naïve and switch groups.

### Statistical analysis

Descriptive statistics were used to summarize demographic and clinical characteristics. Continuous variables were compared using paired t-tests or Wilcoxon signed-rank tests for within-group changes and independent t-tests or Mann-Whitney U tests for between-group comparisons. Categorical variables were analyzed using chi-square or Fisher's exact tests as appropriate. Patients were stratified into two groups for comparative analysis: Naïve group including patients initiating OFA as their first DMT; switch group including patients transitioning from another DMT to OFA.

To further investigate the impact of prior treatments, the switch group was subdivided into patients transitioning from high-efficacy DMTs (fingolimod, NTZ, cladribine, alemtuzumab, and other immunosuppressants [ISs]) and those switching from mild to moderate-efficacy DMTs (dimethyl fumarate, teriflunomide, interferon-beta, and glatiramer-acetate). Differences in treatment outcomes, including relapse rate, MRI activity, and CEW, were compared between these subgroups.

Subgroup analysis was also conducted for patients who had previously discontinued NTZ due to efficacy loss or safety concerns.

To identify factors predictive of response to OFA, a LASSO (Least Absolute Shrinkage and Selection Operator) regression model was applied to assess the relationship between demographic, clinical, and imaging variables and treatment response. The dependent variable was defined as non-response, characterized by sustained MRI activity, relapses, or CEW at T12. The independent variables included age, sex, disease duration, prior DMT exposure, baseline MRI activity (Gd-enhancing lesions and/or new or enlarged T2 lesions), and baseline EDSS. LASSO regression was used to minimize overfitting and select the most relevant predictors. To address class imbalance, oversampling techniques such as SMOTE (Synthetic Minority Over-sampling Technique) were applied. Additionally, model performance was evaluated using the F1-score, which provides a balanced measure of precision and recall. Feature Importance (FI) (range 0–1) was assessed before and after oversampling to evaluate the impact of SMOTE on predictor relevance.

A p-value <0.05 was considered statistically significant. Statistical analyses were conducted using STATA 18.1.

## Results

Out of 263 RMS patients who started OFA in the period between August 2022 and January 2025, a total of 213 patients (135 [62.4 ​%] women, with mean age of 38.9 ​± ​10 years and mean disease duration of 6.1 ​± ​1.9 years), were ultimately enrolled. Of these, 66 (30.9 ​%) were naïve to treatment and 147 (69 ​%) switched from another DMTs. Demographical and clinical characteristics of both cohorts are illustrated in Table 1. Naive patients were younger at the time of the diagnosis with trend toward shorter disease duration compared to patients who switched from other DMTs (Table 1). Moreover, before starting OFA, naïve group had higher number of relapses (3.2 ​± ​1.9 vs 2.4 ​± ​1.5, p ​= ​0.01), and higher number of brain/spine contrast-enhanced lesions (0.2 ​± ​0.1 vs 0.01 ​± ​0.1, p ​= ​0.01) before starting OFA compared to the switch group.

**Table 1:** Demographic and clinical characteristics.

Tot. 213 ​N (%)	Naive 66 (30.9)	Switch 147 (69)	P value
*Female; N (%)*	42 (63.6)	93 (63.2)	0.9
*Age (years); mean ±SD*	37.5 ​± ​9.1	40.2 ​± ​10.6	*0.6*
*Age at onset (years); mean±SD*	29.2 ​± ​7.8	33.4 ​± ​10.2	*0.02*
*Disease duration (years); mean±SD*	5.8 ​± ​1.6	6.4 ​± ​2.4	*0.08*
*EDSS at diagnosis; mean±SD*	2.5 ​± ​1.6	2.9 ​± ​1.9	0.3
*N. of relapses at treatment initiation; mean±SD*	2.6 ​± ​1.7	2.1 ​± ​1.5	0.03

EDSS: Expanded Disability Status Scale; SD; standard deviation.

Overall, after 6 months from OFA initiation, 197 (92.5 ​%) of patients were CEW-free, 188 (88.3 ​%) were relapse-free and 171 (80.3 ​%) were MRI activity-free. At T12, 199 (93.4 ​%) were CEW-free, 198 (93 ​%) were relapse-free and 179 (84 ​%) were MRI activity-free. At T6 after OFA initiation, 162 patients (79.8 ​%) achieved NEDA-3. At T12, the proportion of patients meeting NEDA-3 criteria was 69.5 ​%, 141 patients.

**Fig. 1:**
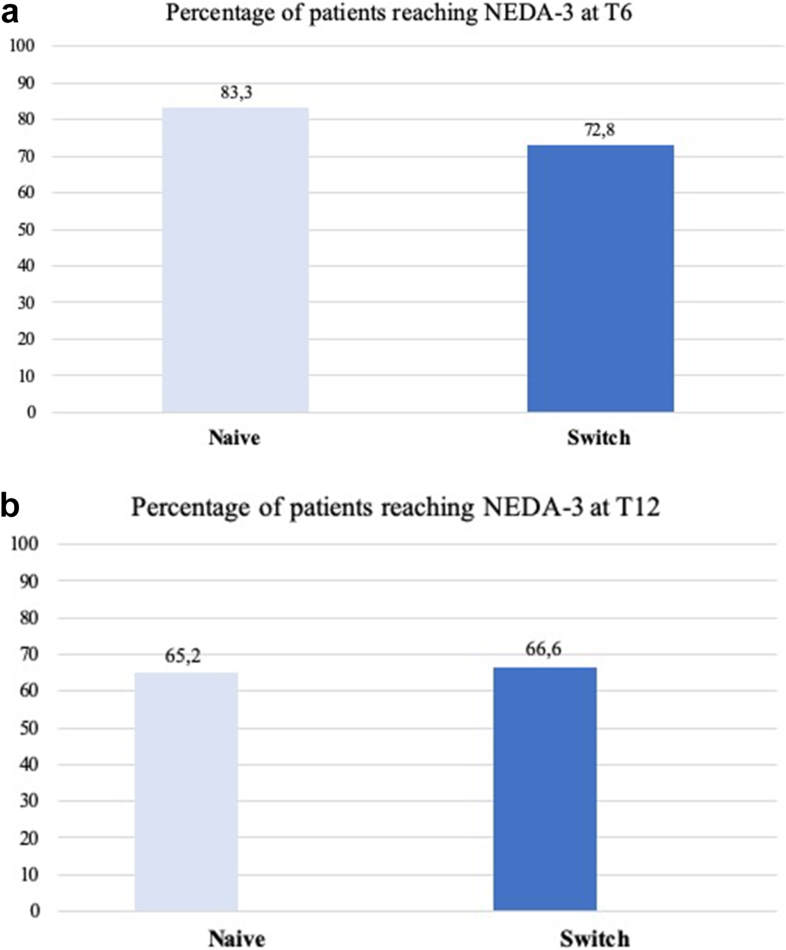
Differences in terms of proportion of patients reaching NEDA-3 between naïve and switch patients at T6 (A) and T12 (B). NEDA-3: non evidence of disease activity.

Both the naïve and switch groups showed not statistically significant differences in EDSS changes within either group over time (p ​= ​0.8 for both groups). On the other hand, a significant reduction in the number of relapses was observed in both groups. In the naïve cohort, the mean relapse rate decreased from 3.2 ​± ​1.9 ​at baseline to 0.6 ​± ​0.9 ​at T6 and further to 0.1 ​± ​0.3 ​at T12 (p ​= ​0.0001). A similar trend was noted in the switch group, with relapses declining from 2.4 ​± ​1.5 ​at baseline to 0.4 ​± ​0.2 ​at T6 and 0.1 ​± ​0.2 ​at T12 (p ​= ​0.0001). Moreover, the number of Gd-enhancing lesions and of new or enlarging T2 lesions showed a marked reduction in both groups. Accordingly, MRI activity was present in 14.2 ​% of naïve patients at baseline, reducing to 12.1 ​% at T12 (p ​= ​0.01). In the switch group, 36.7 ​% exhibited MRI activity at baseline, decreasing to 9.5 ​% at T12 (p ​= ​0.01). No significant differences in terms of efficacy outcomes were found between naive and switch groups (Table 2, Fig. 1A and B). Particularly, at T6, similar proportions of patients were free from CEW (61 (92.4 ​%) vs 135 [91.8 ​%], p ​= ​0.7), relapse-free (58 [87.9 ​%] vs 130 [88.4 ​%], p ​= ​0.8), and MRI activity-free (54 [81.8 ​%] vs 115 [78.2 ​%], p ​= ​0.4), in naïve and switch patients respectively. These trends were maintained at T12, with CEW-free rates of 62 [93.9 ​%] in the naïve group and 138 (93.8 ​%) in the switch group (p ​= ​0.6), relapse-free rates of 61 (92.4 ​%) vs 137 (93.2 ​%) (p ​= ​0.7), and MRI activity-free status in 56 [84.8 ​%] vs 125 (85 ​%) of patients (p ​= ​0.6), respectively.

**Table 2:** Comparison of disability outcomes between treatment-naïve patients and DMT switchers.

Tot. 213 ​N (%)	Naive 66 (30.9)				Switch 147 (69)			
	Baseline	T6	T12	p-value^†^	Baseline	T6	T12	p-value^†^
*EDSS mean±SD*	2.6 ​± ​1.7	2.5 ​± ​1.7	2.4 ​± ​1.3	0.8	2.7 ​± ​1.7	2.6 ​± ​1.5	2.4 ​± ​1.3	0.8
*N. of relapses; mean±SD*	3.2 ​± ​1.9	0.6 ​± ​0.9	0.1 ​± ​0.3	*0.0001*	2.4 ​± ​1.5	0.4 ​± ​0.2	0.2 ​± ​0.3	*0.0001*
*N. of Gd-enhanced lesions; mean±SD*	0.2 ​± ​0.1	0.01 ​± ​0.1	0	*0.03*	0.3 ​± ​0.2	0.01 ​± ​0.1	0	*0.02*
*N. of new or enlarged T2 lesions; mean±SD*	0.8 ​± ​0.3	0.6 ​± ​0.2	0.1 ​± ​0.2	*0.01*	0.5 ​± ​0.7	0.5 ​± ​0.4	0.2 ​± ​0.3	*0.04*
*N of patients with MRI activity (%)*	16 (24.2)	10 (15.2)	8 (12.1)	*0.01*	54 (36.7)	32 (21.8)	22 (15)	*0.01*

DMTs: disease modifying therapies; EDSS: Expanded Disability Status Scale; Gd: gadolinium; SD; standard deviation. MRI activity: presence of Gd-enhanced lesions and/or new or enlarged T2 lesions. ^†^p-values are derived from one-way ANOVA with Bonferroni correction for multiple comparisons.

In the switch group, 42 (28.6 ​% of 147) were previously treated with mild to moderate-efficacy DMTs, while 105 (71.4 ​%) with high-efficacy DMTs. In particular, 20 (47.6%) patients were treated with dimethylfumarate, 14 (33.3%) with teriflunomide, and 8 (19%) with interferons or glatiramer acetate. Among high-efficacy DMTs, 76 (72.4%) patients received NTZ, 15 (14.3%) cladribine tablets, 12 (8.2%) fingolimod, and 2 (1.9%) alemtuzumab. Data about DMTs used before starting OFA treatment in the switch group were reported in the Supplementary Materials (S_Table 1).

**Fig. 2:**
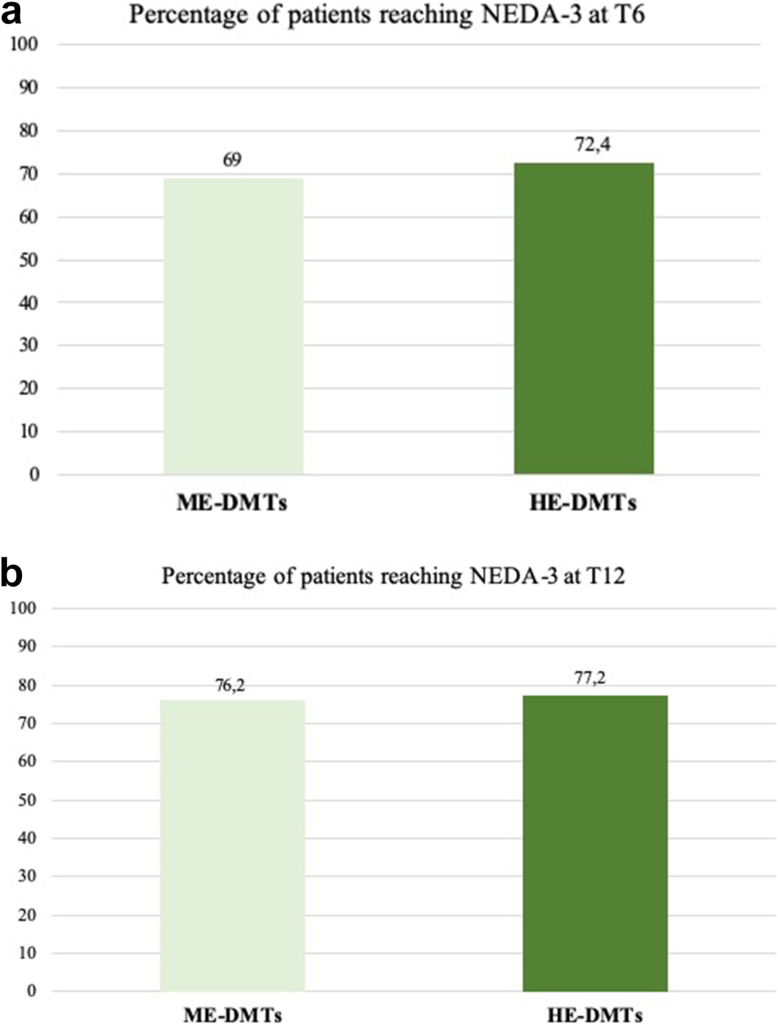
Differences in terms of proportion of patients reaching NEDA-3 between patients previously treated with ME-DMTs and with HE-DMTs at T6 (A) and T12 (B). NEDA-3: non evidence of disease activity, HE-DMTs: high-efficacy disease-modyfing therapies; ME-DMTs: mild to moderate-efficacy disease-modyfing therapies.

The analysis of prior DMTs revealed, at T6, similar proportions of patients were free from CEW (38 [90.5 ​%] vs 97 [92.4 ​%], p ​= ​0.7), free from relapse (36 [85.7 ​%] vs 94 [89.5 ​%], p ​= ​0.7), and from MRI activity (33 [78.6 ​%] vs 82 [78.1 ​%], p ​= ​0.8), in mild to moderate-efficacy DMTs-switchers and high-efficacy DMTs-switchers, respectively. At T12, mild to moderate-efficacy DMTs switchers exhibited a lower relapse rate at T12 compared to those switching from high-efficacy DMTs (0.1 ​± ​0.2 vs 0.3 ​± ​0.4, p ​= ​0.02). In addition, the proportions of patients who remained free from CEW and clinical relapses were comparable between those switching from mild-to moderate-efficacy DMTs and those switching from high-efficacy DMTs. Specifically, CEW-free status was observed in 39 patients (92.9 ​%) vs 99 (94.3 ​%) (p ​= ​0.7), while relapse-free status was seen in 38 (90.5 ​%) vs 99 (94.3 ​%) (p ​= ​0.6), respectively. MRI activity-free rates were higher in the mild to moderate-efficacy switch group compared to high-efficacy group (40 [95.2 ​%] vs 85 [80.6 ​%], p ​= ​0.05) (Table 3). No significant differences were observed in terms NEDA-3 between these groups (Fig. 2 A and B).

**Table 3:** Comparison of disability outcomes between patients switching from mild/moderate- and high-efficacy DMTs.

Tot. 147 ​N (%)	Mild to moderate-efficacy DMTs 42 (28.6)				High-efficacy DMTs 105 (71.4)∗			
	Baseline	T6	T12	p-value^†^	Baseline	T6	T12	p-value^†^
*EDSS mean±SD*	2.6 ​± ​1.4	2.5 ​± ​1.5	2.3 ​± ​1.5	0.8	2.8 ​± ​2.0	2.7 ​± ​1.7	2.5 ​± ​1.1	0.8
*N. of relapses; mean±SD*	2.6 ​± ​1.3	0.6 ​± ​0.2	0.1 ​± ​0.2	*0.0001*	2.2 ​± ​1.7	0.2 ​± ​0.1	0.2 ​± ​0.3	*0.0001*
*N. of Gd-enhanced lesions; mean±SD*	0.4 ​± ​0.2	0.01 ​± ​0.1	0	*0.03*	0.3 ​± ​0.1	0.02 ​± ​0.1	0	*0.02*
*N. of new or enlarged T2 lesions; mean±SD*	0.8 ​± ​0.9	0.6 ​± ​0.4	0.2 ​± ​0.1	*0.01*	0.3 ​± ​0.5	0.4 ​± ​0.3	0.1 ​± ​0.3	*0.04*
*N of patients with MRI activity (%)*	18 (43)	9 (21.4)	2 (4.8)	*0.001*	36 (34.3)	23 (21.9)	20 (19)	*0.001*

DMT: disease modifying therapies; EDSS: Expanded Disability Status Scale; Gd: gadolinium; SD; standard deviation. MRI activity: presence of Gd-enhanced lesions and/or new or enlarged T2 lesions. ∗High-efficacy group includes patients treated with natalizumab and other high-efficacy DMTs (cladribine, fingolimod, alemtuzumab). ^†^p-values are derived from one-way ANOVA with Bonferroni correction for multiple comparisons.

**Fig. 3:**
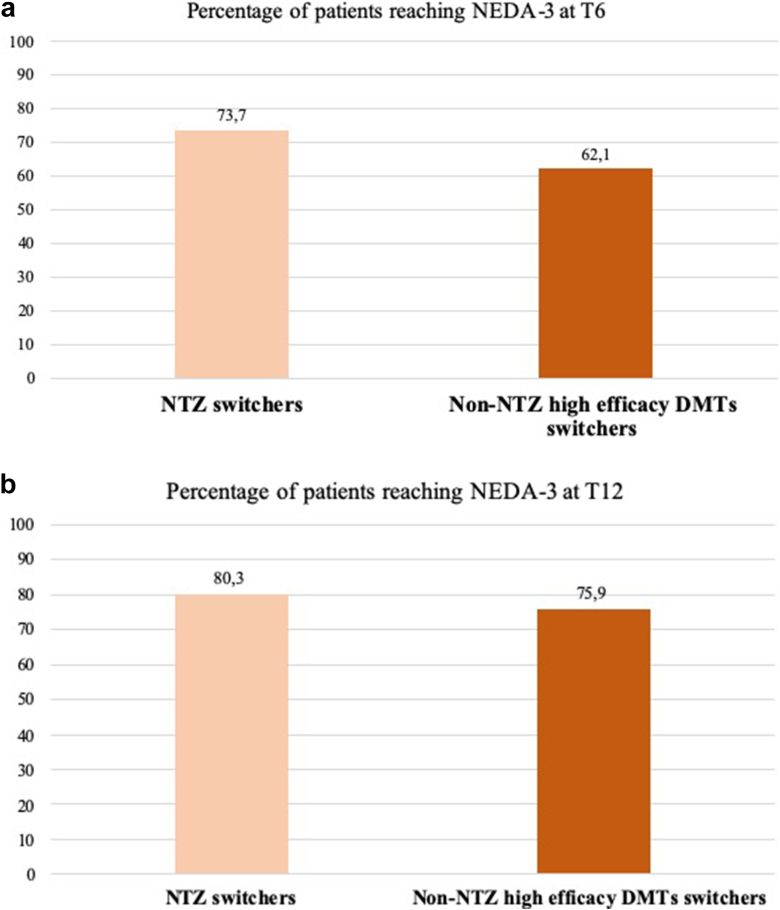
Differences in terms of proportion of patients reaching NEDA-3 between patients previously treated with NTZ and non-NTZ high-efficay DMTs at T6 (A) and T12 (B). NEDA-3: non evidence of disease activity, NTZ: natalizumab; DMTs: disease-modyfing therapies.

Table 4 reports clinical and radiological outcomes in the subgroup of patients who switched from NTZ to OFA (76 patients, 72.4 ​% of the high-efficacy DMT switch group), compared to those who transitioned from other high-efficacy disease-modifying therapies (29 patients, 27.6 ​%). At T6, the proportions of patients free from CEW, from relapses and from MRI activity were similar between the groups (72 [94.7 ​%] vs 25 [86.2 ​%], p ​= ​0.2; 70 [92.1 ​%] vs 24 [82.8 ​%], p ​= ​0.2; 60 [78.9 ​%] vs 22 [75.9 ​%], p ​= ​0.8). After 12 months of OFA treatment, similar rates of patients were free from CEW (73 [96.1 ​%] vs. 26 [89.7 ​%], p ​= ​0.3) and from relapses (63 [82.9 ​%] vs. 24 [82.8 ​%], p ​= ​0.9) in the NTZ and non-NTZ switch groups, respectively. However, NTZ-switchers exhibited significantly higher rates of freedom from MRI activity (69 [90.8 ​%] vs. 16 [55.2 ​%], p ​= ​0.001) compared to those switching from other high-efficacy therapies. No significant differences were observed in terms of NEDA-3 between these groups (Fig. 3 A and B).

**Table 4:** Disability outcomes in patients previously treated with Natalizumab.

Tot. 105 ​N. (%)	NTZ switchers 76 (72.4)				Non-NTZ high-efficacy switchers 29 (27.6)			
	Baseline	T6	T12	p.value^†^	Baseline	T6	T12	p.value^†^
*EDSS mean±SD*	2.2 ​± ​1.1	2.1 ​± ​1.3	2.0 ​± ​1.0	0.8	3.4 ​± ​2.9	3.3 ​± ​2.1	3.0 ​± ​1.2	0.3
*N. of relapses; mean±SD*	0.03 ​± ​0.01	0.1 ​± ​0.02	0.04 ​± ​0.01	0.8	2.3 ​± ​1.7	0.2 ​± ​0.1	0.3 ​± ​0.3	*0.0001*
*N. of Gd-enhanced lesions; mean±SD*	0.4 ​± ​0.2	0.03 ​± ​0.02	0	*0.001*	0.2 ​± ​0.1	0.02 ​± ​0.1	0	*0.001*
*N. of new or enlarged T2 lesions; mean±SD*	0.5 ​± ​0.3	0.6 ​± ​0.2	0.1 ​± ​0.2	*0.08*	0.3 ​± ​0.5	0.5 ​± ​0.3	0.1 ​± ​0.3	*0.07*
*N of patients with MRI activity (%)*	8 (10.5)	9 (11.8)	7 (9.2)	0.6	28 (96.6)	14 (48.3)	13 (44.8)	*0.001*

EDSS: Expanded Disability Status Scale; NTZ: natalizumab; Gd: gadolinium; SD; standard deviation. MRI activity: presence of Gd-enhanced lesions and/or new or enlarged T2 lesions. ^†^p-values are derived from one-way ANOVA with Bonferroni correction for multiple comparisons.

Among NTZ-switchers, a total of 69 (90.8 ​%) patients discontinued NTZ because of high risk of PML, 4 (5.3 ​%) patients because of lack of efficacy, while 3 (3.9 ​%) patients discontinued for other reasons. Those patients discontinuing due to PML risk maintained a higher relapse-free rate at T12 (compared to those discontinuing due to the other subgroups (considering together patients interrupting NTZ because breakthrough disease and other reasons) (58 [84.1 ​%] vs 4 [57.2 ​%], p ​= ​0.01). MRI activity-free survival was also superior in the PML-risk subgroup compared to other subgroups (64 [91.3 ​%] vs 5 [71.4 ​%], p ​= ​0.02).

During the observation period, no serious AEs were reported and all the AEs were mild to moderate. In particular, fever during the first administration was the most frequent AE reported in 153 (71.8 ​% of 213) of treated patients. No significant differences in terms of safety profile were found between naive and switch groups (Supplementary Materials; S_Table 2).

LASSO regression identified baseline MRI activity (β ​= ​0.42, p ​= ​0.003), disease duration >10 years (β ​= ​0.39, p ​= ​0.007), and prior treatment with high-efficacy DMTs (β ​= ​0.45, p ​= ​0.002) as the strongest predictors of non-response to OFA. In contrast, younger age at treatment initiation (β ​= ​−0.37, p ​= ​0.009) was associated with a greater likelihood of response (Table 5). Applying SMOTE oversampling ensured that the model was not biased by the small number of non-responders, improving prediction accuracy. Additionally, model performance was evaluated using the Feature Importance (FI)-score, which provides a balanced measure of precision and recall. The final model achieved a F1-score of 0.79, indicating strong predictive reliability with a strong contribution of the selected features to the model's predictive performance.

**Table 5:** Summary of LASSO model variables.

Variable	Beta Weight (β)	95 ​% CI	p-value
Baseline MRI activity	0.42	(0.15, 0.69)	0.003
Disease duration >10 years	0.39	(0.11, 0.67)	0.007
Prior treatment with high-efficacy DMTs	0.45	(0.22, 0.68)	0.002
Younger age at treatment initiation	−0.37	(-0.62, −0.12)	0.009
Male Sex	0.05	(-0.10, 0.20)	0.21
Baseline EDSS score	−0.12	(-0.35, 0.11)	0.15
Previous treatment with mild-to-moderate efficacy DMTs	0.08	(-0.09, 0.25)	0.18

DMT: disease modifying therapy; EDSS: Expanded Disability Status Scale.

## Discussion

Our study provides real-world evidence supporting the effectiveness and safety of OFA in patients with RMS. The findings reinforce data from pivotal clinical trials while expanding insights into treatment outcomes in a broader patient population, including both treatment-naïve individuals and those transitioning from other DMTs, particularly NTZ. Our results align with several findings, showing a significant reduction in ARR, MRI lesion activity, and disability progression among treated patients [8,19,20]. The observed effectiveness of OFA in our cohort is consistent with pivotal trials such as the ASCLEPIOS I and II studies, which demonstrated superior efficacy of OFA over teriflunomide in reducing ARR and MRI lesion activity [19]. Similarly, our results are also in line with a recent American real-world study on OFA in a total of 175 patients treated with OFA, showing a significant reduction in the number of patients experiencing clinical relapses and of patients with new intracranial T2 lesions and GdE lesions [20].

Additionally, in our cohort, 79.8 ​% of patients achieved NEDA-3 at 6 months post- OFA initiation, 69.5 ​% at 12 months. These findings are consistent with existing literature on OFA and other high-efficacy therapies [19,26,27]. Similarly, in the ALITHIOS study, an open-label extension study assessing the long-term efficacy and safety of OFA over four years, demonstrated high NEDA-3 rates (78.8 ​%) [26]. Comparatively, studies on ocrelizumab have shown that 66.4 ​% of patients with early relapsing-remitting MS achieved NEDA-3 over four years [27].

Furthermore, we did not observe a significant difference in treatment efficacy between naïve patients and those switching from other DMTs, suggesting that OFA provides rapid and consistent benefits regardless of prior therapy. Likewise, the ALITHIOS study showed that patients who switched from teriflunomide to OFA experienced reduced ARR, fewer new or enlarging T2 lesions, and lower serum neurofilament light chain levels, indicating reduced disease activity [26]. The comparative efficacy of OFA against oral DMTs has also been reported in other studies, further supporting its role as a potent therapy for treating relapsing MS [28].

Notably, patients switching from moderately effective therapies showed a slightly better response compared to those previously treated with high-efficacy drugs, in particular for MRI activity. Real-world studies have demonstrated that MRI activity is substantially suppressed one year after initiating OFA [29]. Italian real-world series reported MRI activity in just 3.8 ​% of patients at 12 months (i.e., 96.2 ​% MRI-free) [30]. Although unexpected, our finding may be explained by a lower baseline disease burden or less aggressive disease phenotype in patients switching from moderately effective DMTs. In contrast, those previously treated with high-efficacy therapies likely represent a subgroup with more active, treatment-resistant disease in which OFA appears effective in optimally controlling CEW and preventing relapses, while some residual MRI activity persists, potentially decreasing over longer-term follow-up. In line with this result, residual MRI activity has been reported in patients with high baseline disease activity, typical of those transitioning from high-efficacy DMTs. For example, a Swiss real-world cohort found a significant rate of new lesions within 6 months post-switch from natalizumab, which gradually declined over time [8].

Importantly, our study exhibited the persistence of high efficacy rates across different patient subgroups found in our study, including those transitioning from NTZ [31]. In fact, patients switching from NTZ represent a unique subgroup due to the potential for disease rebound upon discontinuation of NTZ [32,33]. Given NTZ's mechanism of action as an α4-integrin inhibitor that prevents immune cell migration into the central nervous system, it is known that its discontinuation can lead to rapid reactivation of disease activity, manifesting as severe relapses and increased MRI lesion burden [22,32,33]. Similar outcomes have been reported in previous studies evaluating the transition from NTZ to other anti-CD20 therapies, such as ocrelizumab and rituximab, supporting the notion that B-cell depletion can effectively control disease activity following NTZ withdrawal [8,21,31]. Another comparative study examined the transition from NTZ to high-efficacy versus moderate-efficacy therapies, concluding that patients who switched to high-efficacy treatments, such as ocrelizumab or OFA, had a reduced risk of disease reactivation in the first six months post-transition [34]. OFA, in maintaining disease control following NTZ discontinuation, further supporting its utility as a transition strategy [35]. The rapid and sustained B-cell depletion induced by OFA may play a key role in preventing inflammatory reactivation, thereby mitigating the risk of disease rebound [36]. A systematic review evaluating the switch from NTZ to anti-CD20 monoclonal antibodies in patients at risk of PML found that these therapies represent a suitable transition option, with very low clinical relapse rates and a very low incidence of PML [37]. In addition, real-world evidence has highlighted the effectiveness of anti-CD20 therapies, including including OFA, in reducing relapse rates and MRI activity in diverse patient populations [26,29].

Interestingly, patients switching from from other high-efficacy agents showed the highest MRI activity-free rates at 12 months compared to those switching from NTZ. This may reflect differences in the reason for switching; most NTZ patients discontinued due to JCV seroconversion while clinically stable, whereas most of non-NTZ high-efficacy switchers changed therapy due to suboptimal response.

Moreover, our findings suggest that, within the first year of treatment, OFA is well tolerated with a manageable safety profile, with no reports of serious AEs. The most commonly reported AE was fever during the first administration. This is consistent with previously reported infusion- or injection-related reactions associated with anti-CD20 therapies [15,[38], [39], [40]]. Pharmacovigilance studies have further reinforced the safety of OFA, providing comprehensive FAERS data analysis showing a low incidence of severe AEs [41]. Similarly, another study reported a reassuring safety profile, particularly in terms of infection risk and immune-related complications [42]. In support of this, a real-world study investigating safety and efficacy of OFA shortly after its FDA approval during the COVID-19 pandemic demonstrated its feasibility and effectiveness in routine clinical settings [43]. In our cohort, the safety profile of OFA in NTZ-switchers was also favorable, with no evidence of increased AEs compared to other patient subgroups. Notably, none of the patients in our study experienced severe relapses indicative of NTZ rebound syndrome.

Our LASSO regression suggested that patients with a higher inflammatory burden at baseline (as indicated by MRI activity) and those with a long-standing disease course (>10 years) may have a more treatment-resistant disease phenotype. Conversely, younger age at treatment initiation was associated with a higher likelihood of response. This finding aligns with existing evidence suggesting that early intervention in MS is crucial for achieving better disease control and delaying disability progression. Younger patients may have a more treatment-responsive immune system, less cumulative damage, and a greater capacity for recovery, which could explain their improved response rates to OFA.

Although this study offers valuable real-world insights, several limitations should be acknowledged. First, the observational design and relatively short follow-up period limit the ability to evaluate long-term outcomes and sustained treatment effects. Second, the unexpectedly long disease duration observed in treatment-naïve patients may reflect a selection bias inherent to real-world studies, possibly due to delayed diagnosis or deferred treatment initiation. Finally, while the multicenter nature of the study enhances the generalizability of the findings, variability in clinical practice across centers may have influenced patient management and outcome assessments.

In conclusion, the results of this study highlight OFA as an effective and safe treatment option for RMS patients in a real-world setting. The comparable efficacy between naïve and switch patients suggests that OFA can be considered both as a first-choice option both in naïve and switchers patients. Notably, the ability of OFA to prevent disease rebound while maintaining high rates of relapse-free and MRI activity-free status reinforces its role as a viable therapeutic alternative in this challenging patient population. Further real-world research is needed to optimize treatment sequencing strategies and better define the ideal management approach for NTZ discontinuation. Future research should also focus on longer follow-up periods to assess the durability of OFA's benefits, particularly in preventing long-term disability accumulation. Additionally, further studies exploring biomarkers of treatment response may help personalize therapeutic strategies for RMS patients receiving anti-CD20 therapies.

## Informed consent

Each patient participating to the study signed an Informed Consent specifically designed to participate to the study protocol.

## Ethical approval

This study protocol was approved by the local Ethical Committee of the University of Catania (Catania 1, Italy) and by the Ethical Committee of the participating centers. The study was performed in accordance with the ethical standards laid down in the 1964 Declaration of Helsinki and its later amendments.

## Disclosure

Clara G. Chisari reported receiving grants to attend scientific congresses or speaker honoraria from Biogen, Merck-Serono, Novartis, Roche, Sanofi/Genzyme, Teva. Salvatore Lo Fermo, Salvatore Iacono, Giuseppe Schirò, Francesca Ruscica, Sabrina Realmuto, Sebastiano Bucello, Paolo Ragonese, Giuseppe Salemi, Francesca Matta, Simona Toscano, Salvatore Cottone, Luigi Maria Edoardo Grimaldi reported no disclosures.

Francesco Patti reported receiving honoraria for speaking activities by Almirall, Bayer Schering, Biogen Idec, Merck Serono, Novartis, Roche, Sanofi Genzyme, and TEVA; he also served as advisory board member the following companies: Bayer Schering, Biogen Idec, Merck Serono, Novartis, Roche, Sanofi Genzyme, and TEVA; he was also funded by Pfizer and FISM for epidemiological studies; he received grants for congress participation from Almirall, Bayer Shering, Biogen Idec, Merck Serono, Novartis, Roche, Sanofi Genzyme, and TEVA.

## Availability of data and material

Dataset is available under reasonable request to the corresponding author.

## Author contributions

Clara Chisari and Francesco Patti contributed to the study concept and design, to analysis and interpretation of the data and drafted the manuscript. Salvatore Lo Fermo, Salvatore Lo Fermo, Salvatore Iacono, Giuseppe Schirò, Francesca Ruscica, Sabrina Realmuto, Sebastiano Bucello, Paolo Ragonese, Giuseppe Salemi, Francesca Matta, Simona Toscano, Salvatore Cottone, and Luigi Maria Edoardo Grimaldi contributed to acquisition and interpretation of the data and approved the final manuscript.

## Funding

This study was not funded.

## Declaration of competing interest

The authors declare the following financial interests/personal relationships which may be considered as potential competing interests: Clara Grazia Chisari reports a relationship with Sanofi Genzime that includes: funding grants. If there are other authors, they declare that they have no known competing financial interests or personal relationships that could have appeared to influence the work reported in this paper.
