# Supplementary material for: Real-world effectiveness and safety of ofatumumab in relapsing-remitting multiple sclerosis: Insights from naïve and switch patients
Source: Neurotherapeutics. 2025 Sep 15;22(6):e00724. doi: 10.1016/j.neurot.2025.e00724 (PMC12664450; doi:10.1016/j.neurot.2025.e00724)
Supplement: Multimedia component 1 [file mmc1.docx]

**Supplementary Materials**

S_Table 1. Prior treatments, washout duration, and reasons for switching to ofatumumab among patients previously treated with other DMTs.

| Tot. 147 | | **N (%)** | **Wash-out duration**  days; median (IQ) | **Reasons for discontinuation N (%)** | | | |
| --- | --- | --- | --- | --- | --- | --- | --- |
|  |  |  |  | Efficacy | Safety | Other |  |
| Mild to moderate-efficacy DMTs  42 (28,6% of 147 switchers) | *Dimethyl fumarate* | 20 (47.6) | 18 (12-35) | 20 (100) | 0 | 0 |  |
|  | *Teriflunomide* | 14 (33.3) | 22 (16-38) | 14 (100) | 0 | 0 |  |
|  | *IFN/GA* | 8 (19) | 14 (7-23) | 8 (100) | 0 | 0 |  |
| High-efficacy DMTs  105 (71.4% of 147 switchers) | *Natalizumab* | 76 (72.4) | 33 (29-47) | 4 (5.3) | 69 (90.8) | 3 (3.9) |  |
|  | *Cladribine** | 15 (14.3) | 325 (232-653) | 12 (80) | 3 (20) | 0 |  |
|  | *Fingolimod* | 12 (8.2) | 36 (31-44) | 9 (75) | 3 (25) | 0 |  |
|  | *Alemtuzumab*** | 2 (1.9) | 532 (492-743) | 2 (100) | 0 | 0 |  |

*One patient did not complete the full two-course treatment regimen..

**All patients completed both treatment courses.

DMT: disease modifying therapies; IFN/GA: interferons/glatiramer acetate; IQ: interquartile range.

S_Table 2. Safety data.

| Tot. 213  N (%) | **Naive**  **66**  **(30.9)** | **Switch**  **147**  **(69)** | p value |
| --- | --- | --- | --- |
|  |  |  |  |
| Patients reporting at least one AE | 53 (80.3) | 120 (81.6) | 0.8 |
| Detailed AE data |  |  |  |
| *Fever* | 48 (72.7) | 105 (71.4) | 0.8 |
| *Cutaneous reaction* | 2 (3) | 4 (2.7) | 0.8 |
| *Asthenia* | 9 (13.6) | 19 (12.9) | 0.8 |
| *Gastrointestinal symptoms* | 4 (6.1) | 7 (4.8) | 0.7 |

AE: adverse event; DMT: disease modifying therapy
